# Side-by-side 6-mm multi-hole metal stents more effective than inside plastic stents for reintervention after stent-in-stent placement in malignant hilar biliary obstruction

**DOI:** 10.1055/a-2760-9552

**Published:** 2026-01-08

**Authors:** Hirotsugu Maruyama, Kojiro Tanoue, Yuji Kawata, Tatsuya Kurokawa, Yoshinori Shimamoto, Yuki Ishikawa-Kakiya, Yasuhiro Fujiwara

**Affiliations:** 1 12935 Department of Gastroenterology, Graduate School of Medicine, Osaka Metropolitan University, Osaka, Japan

Endoscopic reintervention after the stent-in-stent (SIS) placement of self-expandable metallic stents (SEMSs) for unresectable malignant hilar biliary obstruction (MHBO) is challenging 1 . Regarding revisionary stenting, SEMSs have a longer patency than plastic stents (PSs 2 ), but advances in chemotherapy have increased the need for reinterventions, often using removable PSs. Additional uncovered SEMSs (UCSEMSs) can complicate future reinterventions and cannot be removed, limiting endoscopic options. We report a case of MHBO in which the side-by-side placement of the 6 mm multi-hole SEMS (MHSEMS) was effective for reintervention compared with multiple PSs after SIS.

**Fig. 1:**
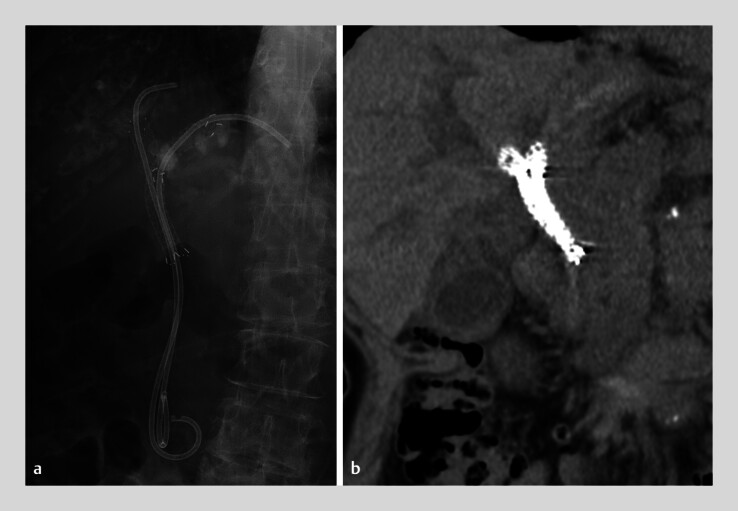
Pre-treatment images. **a** A fluoroscopic image showing plastic stents placed after stent-in-stent deployment of metallic stents for malignant hilar biliary obstruction. **b** A pre-treatment computed tomography image showing dilated intrahepatic bile ducts.

**Fig. 2:**
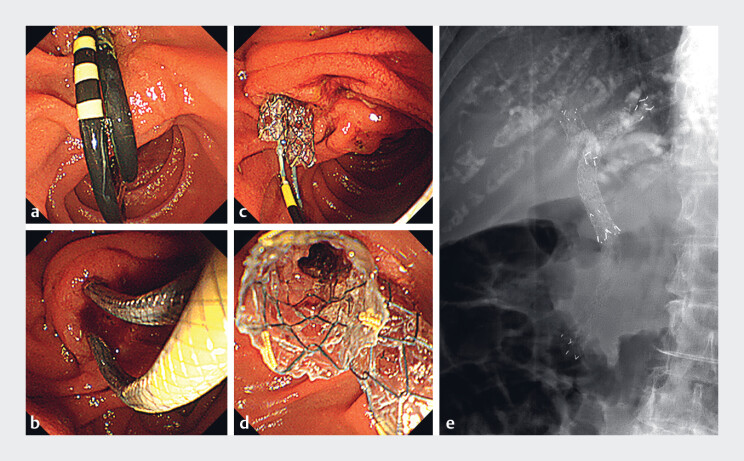
Procedure for reintervention using the side-by-side placement of multi-hole self-expandable metallic stents (MHSEMSs). **a** An endoscopic image showing two plastic stents placed 1 month ago. **b** An endoscopic image showing simultaneous insertion of two MHSEMSs with a slim delivery system into the bile duct. **c** Two MHSEMSs were deployed simultaneously. **d** and **e** Endoscopic and fluoroscopic images after MHSEMS deployment.

An 84-year-old woman with MHBO, previously treated with the MHSEMS and UCSEMS in SIS placement followed by two PSs, was admitted with cholangitis ( Fig. 1 ). She had experienced repeated stent occlusion over a short period, so we opted to place two removable SEMSs ( Video 1 ). A guidewire (GW) was placed into the left intrahepatic bile duct (IHBD), and both PSs were removed. Another GW was placed into the right IHBD, and two 6 mm MHSEMSs with a slim delivery system were inserted. Insertion into the deep left IHBD was somewhat difficult, so the stents were inserted one by one but deployed simultaneously across the papilla ( Fig. 2 ). No adverse events occurred, and the outcome was favorable.

Reintervention using the side-by-side placement of 6 mm multi-hole self-expandable metallic stents after stent-in-stent metal stent placement in malignant hilar biliary obstruction.Video 1

The advantages of the MHSEMS have no step between the delivery system and the stent and it has an outer diameter of 5.9 Fr. In this case, placement of a 7 Fr PS was difficult and time-consuming due to interference from the existing SEMS; however, the MHSEMS could be placed in a much shorter time. Furthermore, after deployment, the inner diameter is larger than that of a PS, and the two MHSEMSs share each other’s holes, providing a drainage effect equivalent to more than 6 mm. The MHSEMS is also removable 3 , making it a favorable option for reintervention.

Endoscopy_UCTN_Code_TTT_1AR_2AZ
